# Who Needs Sex (or Males) Anyway?

**DOI:** 10.1371/journal.pbio.0050099

**Published:** 2007-03-20

**Authors:** Liza Gross

If you own a birdbath, chances are you’re hosting one of evolutionary biology’s most puzzling enigmas: bdelloid rotifers. These microscopic invertebrates—widely distributed in mosses, creeks, ponds, and other freshwater repositories—abandoned sex perhaps 100 million years ago, yet have apparently diverged into nearly 400 species. Bdelloids (the “b” is silent) reproduce through parthenogenesis, which generates offspring with essentially the same genome as their mother from unfertilized eggs. Biologists have yet to find males, hermaphrodites, or any trace of meiosis—the process that creates sex cells—challenging the long-held assumption that evolutionary success requires genetic exchange.

The genetic variation created by meiosis and fertilization, theory holds, bolsters a species’s capacity to weather shifting environmental conditions or resist rapidly evolving parasites. (During meiosis, the genome splits in two, and chromosome pairs swap bits of their DNA; during fertilization, the sex cells fuse to restore the complete genome.) Many multicellular eukaryotes pass through a sexual and asexual phase in their life cycle. But eschewing sex altogether, à la bdelloids, is not theoretically consistent with a long-lived evolutionary life span or extensive species diversification.

In a new study, Diego Fontaneto, Timothy Barraclough, and colleagues developed new statistical techniques for combined molecular and morphological analyses of rotifers to test the notion that species diversification requires sex. The researchers show that, despite an ancient aversion for interbreeding, bdelloids display evolutionary patterns similar to those seen in sexually reproducing taxa. How they have avoided the pitfalls of a lifestyle widely regarded as evolutionary suicide remains an open question.

Bdelloids have remained such an enduring enigma in part because biologists are still debating whether species exist as true evolutionary entities. And if they do, what forces determine how they diverge? Traditional taxonomy relies on morphological differences to classify species, but it can’t distinguish whether such differences reflect physical variations among a group of clones or adaptations among independently evolving populations. In the traditional view of species diversification, interbreeding promotes cohesion within a population—maintaining the species—and barriers to interbreeding (called reproduction isolation) promote species divergence. With no interbreeding to maintain cohesion, the thinking goes, asexual taxa might not diversify into distinct species.

**Figure:**
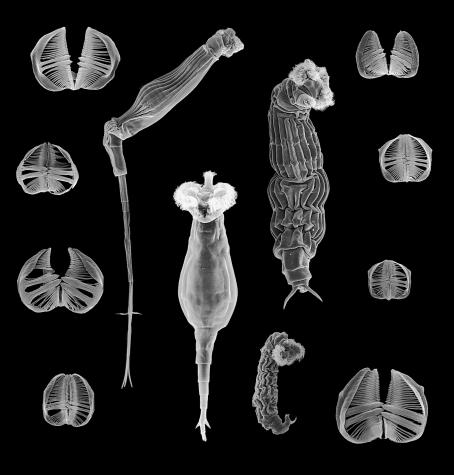
Scanning electron micrographs showing morphological variation of bdelloid rotifers and their jaws. Have these asexual animals really diversified into evolutionary species? (Image: Diego Fontaneto)

Fontaneto et al. defined species as independently evolving, distinct populations (or units of diversity) subject to distinct evolutionary mechanisms. They predicted that if factors other than interbreeding—such as niche specialization—controlled species cohesion and divergence, then asexual taxa should diverge along the same lines as sexually reproducing organisms. And if this were the case, they would expect to find genetic and morphological cohesion within independently evolving populations and divergence between them.

To detect independently evolving populations, the researchers analyzed marker genes isolated from clones of bdelloids collected from diverse habitats around the world. They constructed evolutionary trees using both mitochondrial and nuclear DNA sequences (the molecular “barcode” *cox1*and 28S ribosomal DNA sequences, respectively) to identify species within the samples. For the morphological analysis, they measured the size and shape of the rotifers’ jaws (called trophi).

The morphological results largely fell in line with traditional taxonomic classifications for most bdelloid species. And species identified as related on the DNA trees typically had similar morphology. The correspondence between the molecular and morphological results suggests that the majority of traditionally identified bdelloid species are what’s known as monophyletic—individuals in the same species assort together on the evolutionary tree and share a common ancestor. Only two of these traditional, monophyletic species showed significant variation in trophi size or shape among the populations; both also showed significant divergence in the DNA trees.

Using statistical models to determine the likely origin of the observed DNA tree branching patterns, the researchers show that these distinct monophyletic genetic clusters represent independently evolving entities (rather than variations within a single asexual population). But what caused them to evolve independently? Are they geographically isolated populations that evolved under neutral selection, or did they evolve into ecologically discrete species as a result of divergent selection pressures on trophi morphology?

If bdelloids have experienced divergent selection, the researchers explain, they would expect to see high variation in trophi traits between species, and low intraspecies variation (compared to neutral changes). And that’s what they found—bdelloids have experienced divergent selection on trophi size (and to a lesser degree, on trophi shape) at the species level.

Altogether, these results show that the asexual bdelloids have indeed experienced divergent selection on feeding morphology, most likely as they adapted to different food sources found in different niches. By showing that asexual organisms have diverged into “independently evolving and distinct entities,” the researchers argue, this study “refutes the idea that sex is necessary for diversification into evolutionary species.” They hope others use their approach to study mechanisms underlying species divergence in sexual taxa to clarify the hazy nature of species and biological diversity.

